# SstI Polymorphism of the Apolipoprotein CIII Gene in Iranian Hyperlipidemic Patients: A Study in Semnan Province

**Published:** 2011

**Authors:** Ahmad Reza Bandegi, Mohsen Firoozrai, Mohammad Reza Akbari Eidgahi, Parviz Kokhaei

**Affiliations:** 1 *Department of Biochemistry, Faculty of Medicine, Semnan University of Medical Sciences, Semnan, Iran*; 2 *Department of Biochemistry, Faculty of Medicine, Iran University of Medical Sciences, Tehran, Iran *; 3 *Biotechnology Research Center, Semnan University of Medical Sciences, Semnan, Iran*; 4 *Immune and Gene Therapy Lab, CCK, Karolinska University Hospital Solna, Stockholm, Sweden*; 5 *Department of Immunology, Faculty of Medicine, Semnan University of Medical Sciences, Semnan, Iran*

**Keywords:** Apolipoprotein C-III, Genetic, PCR, Polymorphism

## Abstract

**Objective(s):**

The Sst-I polymorphic site on the 3' untranslated region of the apo CIII gene, has been previously reported to be associated with hypertriglyceridemia. The aim of the present study was to explore the association between Sst-I polymorphism with plasma lipid and lipoprotein levels in hyperlipidemic (HLP) patients from Semnan province, Iran.

**Materials and Methods:**

Genomic DNA was prepared from 76 patients with HLP and 75 matched healthy subjects. DNA samples were amplified by polymerase chain reaction. The samples were analyzed by restriction fragment length polymorphism (RFLP) method using SstI enzyme.

**Results:**

The genotype and allelic frequencies for this polymorphism were significantly different between HLP and normolipidemic groups (*P*< 0.002). Plasma triglyceride (TG) level was higher in both groups, in S2S2 genotype was more than in the S1S1and S1S2 genotypes, however, there was no significant difference in comparison with the control group. Subjects with S1S2 + S2S2 genotypes in compare to S1S1 genotype had odd ratio of 2.8 (95% CI: 1.41-5.56, *P*< 0.003) for developing hypertriglyceridemia.

**Conclusion:**

The results showed that the presence of rare S2 allele was associated with change in TG level in the selected population.

## Introduction

Apolipoprotein CIII (apo CIII), glycoprotein composed of 79 amino acids, is synthesized mainly in the liver (1). In fasting normolipidemic subjects, apo CIII is present on high density lipoproteins (HDL) and triglyceride-rich lipoproteins (TRLs) (2). The *in vivo* function of apo CIII is not clearly described. A number of studies proposed its involvement in the regulation of TRLs catabolism (3,4).* In vitro* studies have demonstrated that apo CIII reduces the catabolism of TRLs by inhibiting lipoprotein lipase (LPL). This enzyme plays a central role in hydrolyzing triglyceride (TG) transported in TRLs (5). Overexpression of human apo CIII gene in transgenic mice resulted in hypertriglyceridemia (6). Apo CIII has direct athrogenic effect on vascular cells (7).

The apo CIII gene is located on the long arm of chromosome 11q23-q24 where it is flanked by the Apo AI and Apo AIV gene; these genes are randomly organized in a cluster of ~17-kb DNA (1). The apo CIII gene contains four exons (8) and is ~ 3.1 kb (3). The expression of apo CIII gene is controlled by positive and negative regulatory elements, which are extended throughout the gene cluster (9).

Several polymorphisms in the apo CIII gene have been detected that associate with variation in plasma lipid concentrations (10-12). One of these genetic variants is SstI polymorphism in the 3´ untranslated region (3´ UTR) of exon 4 in the Apo CIII gene (13,14). This transversion from C to G at nucleotide 3238 results in a loss of the recognition sequence for the restriction enzyme SstI. This substitution produces two alleles: S1 and S2. Multiple studies have suggested a close association between rare S2 allele and elevated levels of plasma TG (15-17) and apo CIII concentration (18,19). Other studies, however, have found no association between the polymorphism and hypertriglyceridemia (20-22).

In view of the importance of apo CIII gene as a marker for hypertriglyceridemia, the present study investigated possible association between SstI polymorphism and plasma lipid levels in 151 individuals from Semnan province, Iran; given the high incidence of dyslipidemia in the Iranian adults (23).

## Materials and Methods

*Study population*

A total of 76 unrelated Iranian subjects (33 males and 43 females) with primary hyperlipidemia were recruited from the (). Subjects with a triglyceride (TG) concentration ≥ 200 mg/dl and Total cholesterol (TC) ≥ 240 mg/dl were included in hyperlipidemic (HLP) group. Patients with secondary hyperlipidemia, hypothyroidism, diabetes mellitus, hypertension, alcoholism, renal failure and hepatic disease as well as patients receiving drugs affecting lipid profile were excluded. Furthermore, 75 age - and sex matched (35 males and 40 females) healthy controls were selected to participate in the study. The healthy control was defined as normal healthy group with plasma concentration of TG <200 mg/dl and TC < 240 mg/dl, and as the one who did not smoke or use lipid - lowering drugs. These subjects were randomly selected by health screening at the same hospital. Both patients and controls had Iranian origin and exhibited a homogenous genetic background. Body mass index () was defined as weight/height² (kg/m²). The study was approved by the local Ethics Committee at Iran University of Medical Sciences and written informed consent was obtained from all subjects participating in the study.

*Plasma lipids and apolipoproteins analysis*

Venous blood samples (10 ml) were obtained from the subjects after an overnight fasting. Total cholesterol, triglycerides, high density lipoprotein cholesterol (HDL-c), and low density lipoprotein-cholesterol (LDL-c) were determined enzymatically. ApoAI and apoB levels were measured by immunoturbidometric method. All biochemical tests were performed in serum by COBAS MIRA analyzer and commercial kits (Pars Azmon Co. Iran).

*Genetic analysis*

**Figure 1.**
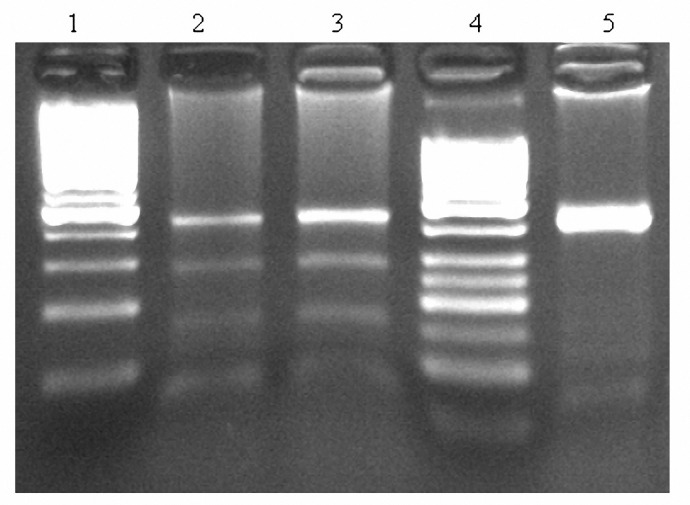
Restriction patterns of SstI site Lanes 2 and 3 are from individuals with S1S2 genotype (The sizes of bands are 428, 269 and 159 bp). Lane 5 is from an individual with S1S1 genotype. Lanes 1 and 4 show the DNA size markers (100 bp and 50 bp , respectively). Agarose 2%, TBE buffer and staining with EB

**Figure 2.**
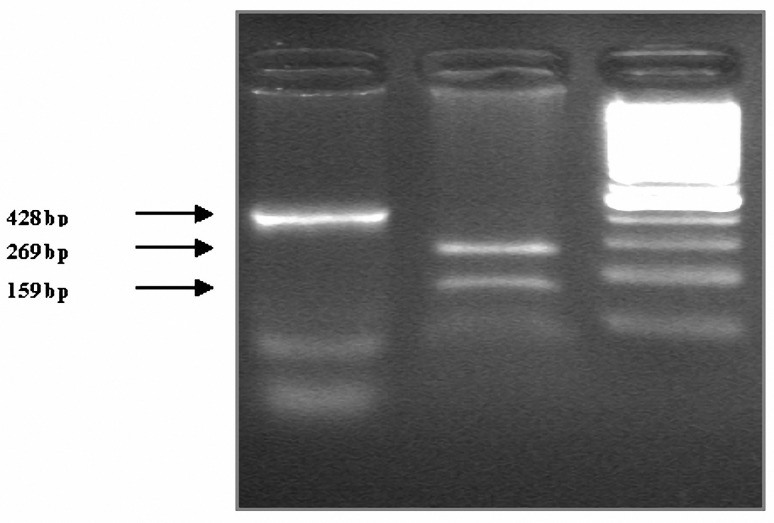
Restriction patterns of SstI site. Lane 1 is from individual with S1S1 genotype. Lane 2 is from an individual with S2S2 genotype. Lane 3 shows the DNA size markers (100 bp). Agarose 2%, TBE buffer and staining with EB

Genomic DNA was isolated from blood leukocytes, using a "salting out" procedure (24). A fragment of 428 bp in the apo C III 3´-untranslated region (UTR) of exon 4 containing the polymorphic SstI site, amplified by Polymerase chain reaction (PCR). The primers (25) used were: F: 5' - GGT GAC CGA TGG CTT CAG TTC CCT GA-3' and R: 5'- CAG AAG GTG GAT AGA GCG CTG GCC T-3'(DNA Technology A/S (). All reactions were performed in 25 μl reaction volumes containing 100 pmol of each primer, 50 ng genomic DNA, 2.5 μl of 10x reaction buffer, 2 mM MgCl2, 0.1 mM dNTP and 1 unit Taq DNA polymerase (Roche Diagnostics GmbH, ). The PCR conditions were: 1 cycle at 95 °C for 5 min, 35 cycles at 95 °C for 1 min and 1 min at 64 °C, with a final elongation at 72 °C for 5 min. Five μl of the PCR product were digested at 37 °C for 4 hr with 2.5 units of SstI restriction enzyme (Roche Diagnostics GmbH, Germany) and specific restriction buffer in a final volume of 25 μl. Following digestion, the samples were run on 2% agarose gel and visualized directly over a UV transilluminator. Alleles were defined as S1 and S2 based on the absence or presence of the SstI restriction site, respectively. The presence of the SstI site (S2 allele) resulted in two fragments of 269 and 159 bp (Figures 1 and 2).

*Statistical analysis*

Continuous variable were reported as mean±SEM (standard error of the mean). Analysis of variance (one-way ANOVA) and student's t-test were used for the comparison of lipid parameters means among the various genotypes. Hardy-Weinberg equilibrium was tested by the X² test. The distribution of genotype and allele frequencies between hyperlipidemic subjects and the control group were compared using Pearson's chi-square test. Statistical significance was accepted at *P*< 0.05. Statistical analysis was performed with SPSS10/win statistical software.

## Results

Total cholesterol (TC), TG, LDL-C and apo B were significantly (*P*< 0.001) higher in primary hyperlipidemia cases in compare to the control subjects. The hyperlipidemic subjects also had significant decrease (*P*< 0.001) levels of HDL-c and apoAI compared to the control subjects. The control and patient groups did not differ significantly with respect to age and sex (Table 1).

The distribution of SstI genotypes and alleles frequencies among healthy subjects and hyperlipidemia patients are shown in Table 2. There was significant difference in the genotypes S2 S2 distribution (*P*< 0.01) and rare S2 allele (*P*< 0.002) between the two groups. The distribution of genotypes observed in both groups was in Hardy-Weinberg equilibrium (Table 3).

**Table 1.** General characteristics and lipid profile (mg/dl) of the study population

Variable	Control (n= 75)	Patient (n= 76)
Age (year)	45.3±1.4	46.1 ± 1.2
Men/women	35/40	34/42
BMI( kg/m²)	24.7±0.4	26.9±0.4 *
TC	131±5	298±6 *
TG	166±4	430±10 *
LDL-c	68±3	154±5 *
HDL-c	45.4±0.8	35.7±0.8 *
Apo AI	138±2	119±2*
Apo B	48±1.2	114±3*

Data present the mean±SEM; *P< 0.001 compared to the control, BMI= Body mass index, TC= Total cholesterol,TG= Triacylglycerol, LDL-c= Low density lipoprotein – cholesterol, HDL-c= High density lipoprotein – cholesterol, ApoAI= Apolipoprotein AI, ApoB = Apolipoprotein B

**Table 2.** Genetic distribution and allelic frequencies for SstI polymorphism in the subject groups

Genotype	Control n (%) n= 75	Patient n (%) n= 76	*P* Value
S1S1	56 (74.7)	39 (51.3)	
S1S2	17 (22.7)	31 (41)	<0.002
S2S2	2 (2.7)	6 (8)	
Allele frequency			
S1	86	71.7	< 0.01
S2	14	28.3	

**Table 3.** Frequency distribution of various genotypes and alleles of Apo CIII SstI polymorphism *

Genotype	Frequency observed genotype (n)	Expected genotype frequency	Allele frequency
S1S1	95	93.8	S1 = 0.79
S1S2	48	50.4	S2 = 0.21
S2S2	8	6.8	
Total (n)	151		

*Test for Hardy-Weinberg equilibrium: Chi-square= 0.34, df= 2, *P*> 0.1

**Table 4.** Characteristic of patient group according to different genotypes of SstI polymorphism

Variable	S1S1	S1S2	S2S2
N	39	31	6
Age (year)	46±1.7	46.9±1.9	43±5.1
Men/women	22/17	10/21	2/4
BMI (kg/m²)	26.7±3.7	27.2±0.6	25.9±2.3
TC (mg /dl)	293 ± 9	297 ± 9	309 ± 27
TG (mg /dl)	379 ± 12	468 ± 8	560 ± 38*
LDL-c (mg/dl)	151 ± 6	157 ± 8	153 ± 5
HDL-c (mg/dl)	36.2 ± 0.3	35.4 ± 0.6	34 ± 1.2
LDL-c/HDL-c	4.2± 0.4	4.6± 0.7	4.5± 0.5
Apo AI (mg/dl)	121 ± 2	118 ± 3	117 ± 6
Apo B (mg/dl)	166 ± 8	172 ± 6	163 ± 6

Data present the mean ± SEM; *P< 0.001 compared to the control,BMI = Body mass index, TC= Total cholesterol, TG= Triglyceride, LDL-c= Low density lipoprotein – cholesterol, HDL-c= High density lipoprotein – cholesterol, Apo-AI= Apolipoprotein AI, ApoB= Apolipoprotein B

**Table 5.** haracteristic of the control group according to different genotypes of SstI polymorphism

Variable	S1S1	S1S2	S2S2
N	56	17	2
Age (year)	47±1.5	42±3.5	38±4
Men/women	27/29	7/10	1/1
BMI (kg/m²)	24.2± 0.4	25.75±0.9	25.8± 1.3
TC (mg /dl)	132± 5.1	126 ± 11.5	144 ± 39
TG (mg /dl)	153 ± 3	156 ± 7	160 ± 25
LDL-c (mg/dl)	71 ± 3.8	58.4 ± 4	64.5 ± 19
HDL-c (mg/dl)	44.7 ± 0.8	44.2 ± 2	51 ± 10
Apo AI (mg/dl)	137 ± 2	141 ± 4	137 ± 9
Apo B (mg/dl)	97 ± 4	94 ± 9	105 ± 24

Data present the mean±SEM; *P< 0.001 compared to the control, BMI= Body mass index, TC= Total cholesterol, TG= Triglyceride, LDL-c= Low density lipoprotein – cholesterol, HDL-c= High density lipoprotein – cholesterol, Apo-AI= Apolipoprotein AI, ApoB = Apolipoprotein B

**Table 6.** Pearson correlation coefficients between SstI polymorphism and other parameters

	Control group			Patients group	
	
	r	*P*		r	p
Age	0.181	0.120		-0.010	0.929
Sex	-0.053	0.650		-0.130	0.265
BMI	-.0.171	0.143		-0.045	0.697
TC	-0.041	0.721		-0.015	0.895
TG	-0.247	0.032*		-0.363	0.001**
LDL-c	0.195	0.093		0.07	0.577
HDL-c	-0.171	0.141		-0.157	0.175
Apo AI	-0.110	0.347		-0.152	0.190
Apo B	0.019	0.872		-0.074	0.528

*Correlation is significant at the o.o1 level (2-tailed); **Correlation is significant at the o.o1 level (2-tailed), BMI= Body mass index, TC= Total cholesterol, TG = Triglyceride, LDL-c = Low density lipoprotein – cholesterol, HDL-c= High density lipoprotein – cholesterol, Apo-AI= Apolipoprotein AI, ApoB= Apolipoprotein B

The lipid and apolipoprotein levels of different genotypes of the HLP and control groups are shown in Tables 4 and 5, respectively. TG was significantly different among various genotypes in the HLP (*P*< 0.001) and control (not significant) groups. In particular, the concentration of TG was at the highest level in the S2S2 subjects followed by S1S2 and then by S1S1 in the HLP and control groups.

As it has been shown in Tables 4 and 5, no significant differences were observed in TC, LDL-c, HDL-c, apoAI, apoB levels of various genotypes of SstI in both groups.

In the HLP and control groups (Table 6), SstI polymorphism (Presence of S2 allele) correlated significantly with TG (r=-0.247 and r= -0.363 in the control and patients groups, respectively).

The crude odd ratio (OR) for S1S2 + S2S2 genotypes (in comparison to S1S1 genotype) was found to be 2.8 (95% CI: 1.41-5.56, *P*< 0.003), which was highly significant.

## Discussion

The prevalence of dyslipidemia in Iranian adults is relatively high. Azizi *et al *(23) reported higher levels of TGs, TC and LDL-c and slightly lower levels of HDL-c in Iranian adults in compare to studies from industrialized countries. In addition to environmental factors, genetic factors play an important role in determining lipid plasma levels (26). Some epidemiological studies have shown associations between polymorphisms in apo AI-CIII-AIV cluster gene and variation in plasma lipids concentration (27-29). In addition, many of these studies have been carried out on the SstI polymorphism in the 3´ UTR of apo CIII gene (12,30).

Some studies have showed a large difference in the frequency of the rare S2 allele among races, especially in Caucasians and Non-Caucasians. Although, the frequency of S2 allele in Caucasians has been reported to be 0.16 and 0.09 in hypertriglyceridemic (HTG) and normotriglyceridemic (NTG) individuals (15). The frequencies were higher for different Asian populations (16, 31-35) and 0.28 and 0.14 in the current study.

The observed frequency of S2 allele is higher in Asian populations than in Caucasians. Our data indicated that S2 frequency is higher in Iranian (Middle East) than in Caucasians, and is lower than the S2 frequency reported form region.

We observed that SstI polymorphism was significantly correlated with plasma TG levels. 

Our findings were in line with previous studies, which explained a significant SstI polymorphism and TG levels (32,36). 

However, these findings have not been confirmed by other investigators (30,37). In the present study, S2S2 individuals showed the highest levels of TG followed by S1S2 and S1S1 in HLP (*P*< 0.001) and the control groups.

The molecular bases by which the SstI polymorphism can influence the level of TGs are still unknown. Since the SstI polymorphism is located in the non-coding region of exon 4 of the apo CIII gene; it is believed that the S2 allele is in linkage disequilibrium with one or more sequences within or nearby the apo CIII gene, affecting TG concentration. Dammerman *et al* (13) reported that the SstI polymorphism is in linkage disequilibrium with mutations of -625 and -482 in the apo CIII promoter region.

There was no evidence for statistically significant association between SstI polymorphism of the apo CIII gene and TC, HDL-c or LDL-c in the current study. These findings were in agreement with the previous studies (35,37).

## Conclusion

The presence of S2 allele is shown to be associated with high TGs levels in HLP (significant) and normal (not significant) subjects.

Since the current research has been performed in a restricted area and also there are many ethnic groups in , we suggest further studies to be performed on larger populations on different ethnic groups. 

Considering the fact that there is a high prevalence of coronary artery disease (CAD) among the Iranian population (38), and also dyslipidemia, including high triglyceride level is a risk factor for CAD, studies investigating the relationship between apo CIII SstI polymorphism and CAD seem necessary to be carried out in Iran.
